# Salvage Procedure in Case of Urethrocavernous Fistula after Revision Surgery for Malfunctioning Three-Piece Penile Prosthesis

**DOI:** 10.1155/2016/4179862

**Published:** 2016-01-27

**Authors:** Enrico Caraceni, Angelo Marronaro, Luca Leone

**Affiliations:** U.O. Urologia Civitanova Marche, Area Vasta 3 Asur Marche, Via dei Ginepri, 62012 Civitanova Marche, Italy

## Abstract

Urethrocavernous fistula is a rare complication of penile prosthesis. Literature lacks any materials regarding this complication's treatment. We report our experience with a 66-year-old man who developed urethrocavernous fistula after penile prosthesis implant. Our technique involves the careful closure of urethral and corpus cavernosum defects with application of TachoSil^*®*^ above the sutures. After the salvage procedure, no recurrence of fistula occurred and patient was able to have sexual intercourse. We believe that our technique may be successfully used in case of urethrocavernous fistula after penile prosthesis implant.

## 1. Introduction

Nowadays, penile prosthesis implant represents the definitive treatment of erectile dysfunction in those patients in which phosphodiesterase type 5 inhibitor, intracorporeal injection, and/or vacuum device are ineffective [1].

Three-piece inflatable prosthesis, mostly with an antibiotic coating, is the preferred choice [2], although some urologists still prefer malleable prosthesis as being less expensive and easier to implant than inflatable ones. It has been proved that hydraulic three-component prosthesis improves the quality of life of the patient with severe ED [3].

Intraoperative complications of inflatable prosthesis involve the urethra, the bladder, and intestine. Breakage of the device is also a possible complication during implantation.

Urethral injuries may occur during dilatation of corpora cavernosa and can especially affect scared tissue. Bladder and visceral injuries can happen during the positioning of the reservoir especially with the penoscrotal incision.

Postoperative complications include infection (1.7–15%) and mechanical disorders of the prosthesis (fluid loss, cylinder rupture, and mechanical breakage) with a percentage ranging from 1.4% to 11% [4, 5].

Most of these complications require removal of the prosthesis.

We present a case of urethral injuries which occurred during a penile prosthesis implant followed by urethrocavernous fistula which developed one month after the surgery.

## 2. Case Presentation

The patient is a 66-year-old man with a history of diabetes, obesity, high blood pressure, and erectile dysfunction nonresponsive to both systemic and local treatment. For this reason, he underwent a 3-piece Inflatable Penile Implant in 1996 in another centre. Because of prosthesis malfunction, he had revision surgery of the implant in 2000 and 2004 in the same centre. Patients had been using implant up to 2007. Subsequently, the device had a mechanical failure and patient was not able to have sexual intercourse anymore.

In 2009, he underwent a radical prostatectomy for prostate cancer.

As mentioned above, penile prosthesis was not functioning. As a consequence, the reservoir was removed while cylinders and scrotal pump were left implanted in the penis. In April 2013, he underwent a revision surgery of the nonfunctioning system on the patient's request.

**Figure 1:**
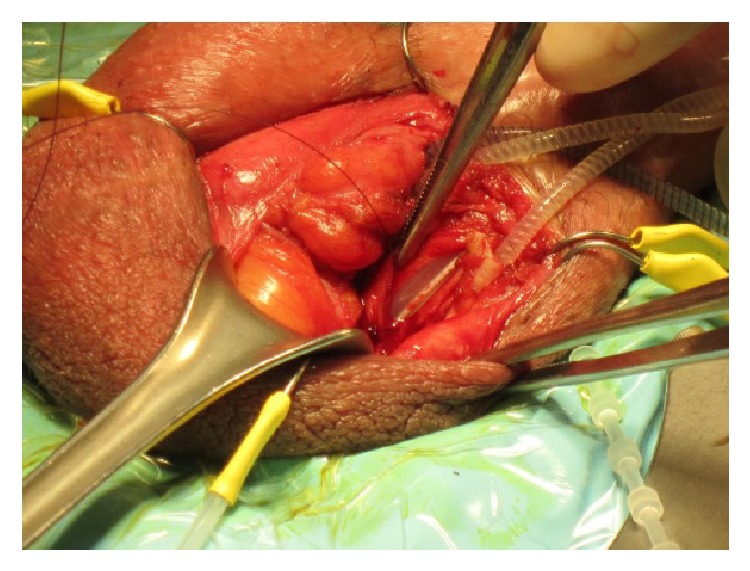
Intraoperative image showing the urethral lesion.

A transverse skin incision at the penoscrotal junction was made and the cylinders-reservoir connecting tube was isolated up to internal inguinal ring. An artificial erection was inducted using saline solution. The manoeuvre showed a nonlasting erection, suggesting a water loss from the cylinders. For this reason, we decided to remove both the pump and the cylinders and make a new implant. A 2 cm corporotomy was made bilaterally and cylinders were removed. Unfortunately, a 3 cm length urethral lesion occurred during the isolation of connecting tubes between pump and left cylinder by electric cautery knife. Urethral catheter was evident through the lesion as shown in Figure 1.

Strict adherence between connecting tubes and corpus spongiosum of urethra may have facilitated the occurrence of the lesion. We decide to close the urethral defect with a monocryl 4/0 continuous suture. Implantation of a new AMS 700, 3-piece inflatable penile prosthesis was immediately performed.

At the end of the procedure, a sovrapubic catheter was inserted. Urethral catheter was also left in situ.

**Figure 2:**
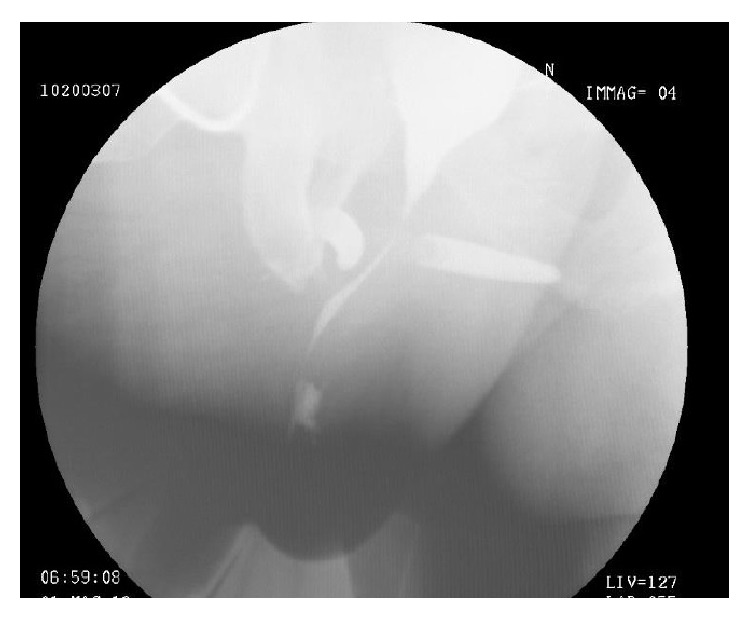
Voiding cystourethrogram showing radiocontrast leak.

On May 2013, 30 days after the procedure, a voiding cystourethrogram showed a radiocontrast leak suggesting a persistence of urethral lesion one month after the implant (Figure 2); the retrograde urethrography was avoided for the infection risk.

We suspected the presence of an urethrocavernous fistula because the lesion was at the penoscrotal junction level, the same level of corporotomy. Fistula strongly increases the risk of infection of penile prosthesis. In spite of this, we decided to perform a salvage procedure in order to repair the fistula trying not to remove the implant.

**Figure 3:**
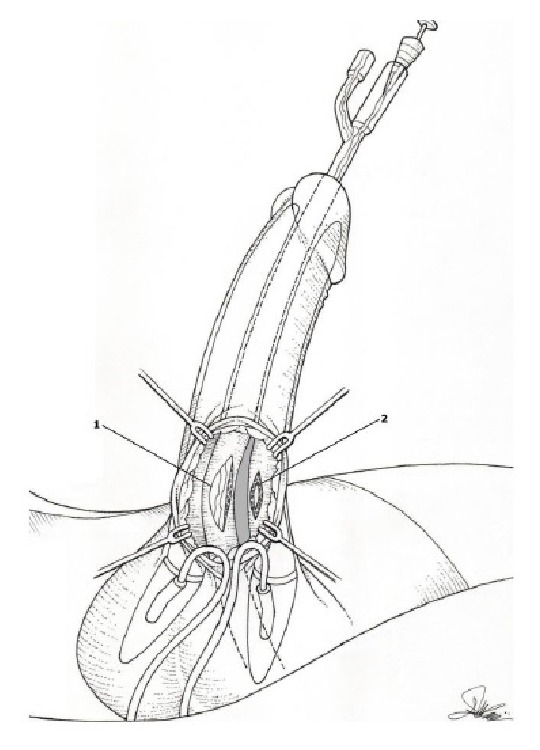
Urethral (1) and left corpus cavernosum (2) defects after fistula separation.

First step was an accurate isolation of the urethra up to the bulbar portion and its separation from corpora cavernous to the urethrocavernous fistula was identified. The maneuver resulted in the separation of the fistula. Length of urethral and left corpus cavernous defect was about 2 cm and 1 cm, respectively. Defects were located at the penoscrotal angle (see Figure 3).

After wound and prosthesis washing with different antiseptic solutions including antibiotics, hydrogen peroxide, and betadine [5], we closed the urethra and corpus cavernosum defect by 4/0 PDS double layer suture and 3/0 monocryl suture, respectively.

With the aim of preventing fistula recurrence, we decided to use a patch sponge coated with a dry layer of the human coagulation factors fibrinogen and thrombin (TachoSil) [6].

TachoSil is known to promote haemostasis and tissue sealing quickly and easily.

We covered both left corpus cavernosum and urethral suture by applications of TachoSil. Patch length was 2 × 2 and 2 × 3 cm, respectively.

**Figure 4:**
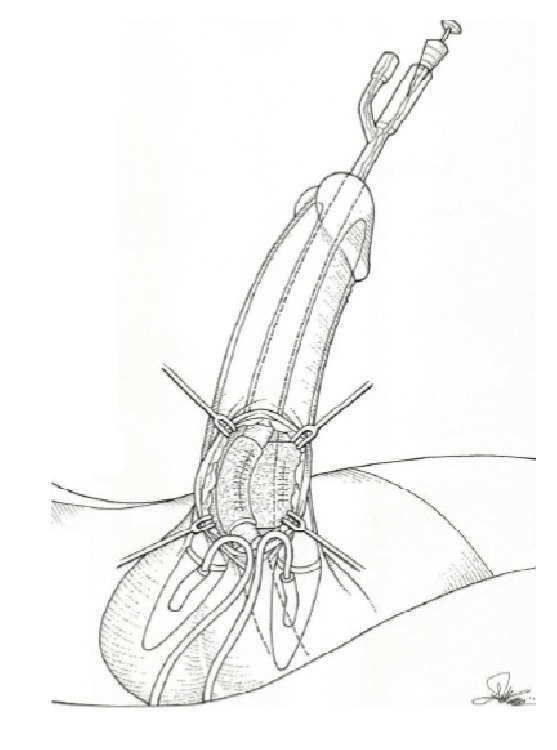
TachoSil patch covering urethral and left corpus cavernosum defects.

Another patch sponge (2 × 3 cm) was applied around the corpus spongiosum just above the sutured defect (see Figure 4).

After accurate washing with antiseptic solutions, the wound was closed in layers. Dartos fascia and skin were both closed with absorbable suture. A 14 Ch sovrapubic and urethral catheter were left in situ.

One month later, a retrograde urethrocystography showed no signs of fistula communicating urethra and left corpus cavernosum.

Sovrapubic and urethral catheter were removed. Patient was allowed to activate penile prosthesis and to have sexual intercourse. No complications or fistula recurrence occurred after the described salvage procedure. Up to now (16 months of follow-up), penile prosthesis is still functioning.

## 3. Discussion

Urethral perforation is a rare intraoperative complication of prosthesis implant. It is usually a consequence of incorrect dilators introduction. Risk of urethral injury is increased in patients with extensive fibrosis of the penis [7, 8]. There is still debate about the optimal management of this complication. The treatment option is urethral repair for proximal perforations. If the perforation involves the urethral meatus, the implant of penile prosthesis should be abandoned and postponed [6]. Urethral and suprapubic catheter may help the closure of the perforation. It is also possible to delay insertion of cylinders or implant a malleable prosthesis until the damaged urethra has healed. The malleable prosthesis will be replaced by the inflatable one at a later date during a second operation. Urethrocavernous fistula is an even more infrequent complication of a penile prosthesis. It was only reported as a rare complication after sexual intercourse, after penile fracture, and after cavernospongiosum shunt for priapism [9–11]. To our knowledge, urethrocavernous fistula after penile prosthesis implant was described only once by García et al. [12]. In that case, prosthesis was removed and corpus cavernosum was sutured.

To our knowledge, this is the first reported salvage procedure for urethrocavernous fistula developed after revision surgery for malfunctioning penile prosthesis.

As described above, urethral lesion occurred during isolation of connecting tubes by electrocautery knife. We only closed the defect by suturing. After that, we proceeded to a new implant. Urethrocavernous fistula occurred a month after the procedure despite the fact that both sovrapubic and urethral catheter were left in situ. This means that suturing without further maneuvers except the urinary drainage may be not sufficient to prevent this complication. Fistula occurrence might possibly have been avoided if we had stopped the operation without implanting a new prosthesis or by complete isolation of urethra and immediate application of TachoSil.

In addition, patient had been complaining of moderate urinary incontinence due to the previous radical prostatectomy. In fact, he reported urine leakage around the catheter between catheter and urethra during Valsalva maneuver after the first procedure. As a consequence, urethral suture was not completely dry and this could have facilitated fistula arising.

On the contrary, complete isolation of corpus spongiosum, careful suturing of urethral and corpus cavernous defects, and application of TachoSil to reinforce sutures allowed us to repair fistula and avoid its recurrence.

Drainage of urine by sovrapubic and urethral catheter is also an essential step.

## 4. Conclusions

We believe that technique described in this salvage procedure may be used in case of urethrocavernous fistula after insertion of penile prosthesis.

In particular, we were able to avoid fistula recurrence without removing the implant.
